# Brain Areas Controlling Heart Rate Variability in Tinnitus and Tinnitus-Related Distress

**DOI:** 10.1371/journal.pone.0059728

**Published:** 2013-03-22

**Authors:** Sven Vanneste, Dirk De Ridder

**Affiliations:** 1 Brai^2^n, Tinnitus Research Initiative Clinic Antwerp & Department of Neurosurgery, University Hospital Antwerp, Antwerp, Belgium; 2 Department of Translational Neuroscience, Faculty of Medicine, University of Antwerp, Antwerp, Belgium; University of Adelaide, Australia

## Abstract

**Background:**

Tinnitus is defined as an intrinsic sound perception that cannot be attributed to an external sound source. Distress in tinnitus patients is related to increased beta activity in the dorsal part of the anterior cingulate and the amount of distress correlates with network activity consisting of the amygdala-anterior cingulate cortex-insula-parahippocampus. Previous research also revealed that distress is associated to a higher sympathetic (OS) tone in tinnitus patients and tinnitus suppression to increased parasympathetic (PS) tone.

**Methodology:**

The aim of the present study is to investigate the relationship between tinnitus distress and the autonomic nervous system and find out which cortical areas are involved in the autonomic nervous system influences in tinnitus distress by the use of source localized resting state electroencephalogram (EEG) recordings and electrocardiogram (ECG). Twenty-one tinnitus patients were included in this study.

**Conclusions:**

The results indicate that the dorsal and subgenual anterior cingulate, as well as the left and right insula are important in the central control of heart rate variability in tinnitus patients. Whereas the sympathovagal balance is controlled by the subgenual and pregenual anterior cingulate cortex, the right insula controls sympathetic activity and the left insula the parasympathetic activity. The perceived distress in tinnitus patients seems to be sympathetically mediated.

## Introduction

Tinnitus is defined as an intrinsic sound perception that cannot be attributed to an external sound source. This phantom perception is a common disorder. The American Tinnitus Association estimates that 50 million Americans are affected by it, and that 12 million of these people seek medical attention because of their tinnitus [1]. In about 6 to 25% of the affected people tinnitus causes a considerable amount of distress [2]–[4], resulting in about 2–4% of the population who are severely impaired in their quality of life [5]. Tinnitus can interfere with sleep and concentration, social interaction and work [6]. Increased prevalence rates of anxiety and depression are reported among tinnitus patients [7], [8].

Distress in tinnitus patients is related to increased beta activity in the dorsal part of the anterior cingulate and the amount of distress correlates with an EEG alpha network activity consisting of the amygdala-anterior cingulate cortex-insula-parahippocampus as demonstrated both by source analysis of Fourier based data [9] and independent component analysis [10]. Using MEG, long-range coupling between frontal, parietal and cingulate brain areas in alpha and gamma phase synchronization has been shown to be related to tinnitus distress [11]. The distress in tinnitus patients also correlates with an increase in incoming and outgoing connections in the gamma band in the prefrontal cortex and the parieto-occipital region [12].

Adaptation under conditions of stress is a priority for all organisms. Stress can be broadly defined as an actual or anticipated disruption of homeostasis or an anticipated threat to well-being [13]. Stressor-related information from all major sensory systems is conveyed to the brain, which recruits neural and neuroendocrine systems (effectors) to minimize the net cost to the animal. The physiological response to stress involves an efficient and highly conserved set of interlocking systems and aims to maintain physiological integrity even in the most demanding of circumstances [13].

The autonomic nervous system provides the most immediate response to stressor exposure - through its sympathetic and parasympathetic arms, which provoke rapid alterations in physiological states through neural innervation of end organs. The autonomic nervous system is a collection of afferent and efferent neurons that link the central nervous system with visceral effectors. The two efferent arms of the autonomic nervous system - the sympathetic and parasympathetic arms - consist of parallel and differentially regulated pathways made up of cholinergic neurons (preganglionic neurons) located within the central nervous system that innervate ganglia (for example, para- or pre-vertebral sympathetic ganglia), glands (adrenal glands) or neural networks of varying complexity (enteric or cardiac ganglionic networks). These peripheral ganglia and networks contain the motor neurons (ganglionic neurons) that control smooth muscles and other visceral targets. The sympathetic ganglionic neurons that control cardiovascular targets are primarily noradrenergic [14]. The sympatho-adrenomedullary arm can rapidly (in seconds) increase heart rate and blood pressure by exciting the cardiovascular system. Importantly, excitation of the autonomic nervous system wanes quickly - owing to reflex parasympathetic activation - resulting in short-lived responses [13]. Previous research also revealed that distress is associated to a higher sympathetic (OS) tone in tinnitus patients [15] and tinnitus suppression to increased parasympathetic (PS) tone [16]. The heart is dually innervated by the autonomic nervous system such that relative increases in sympathetic activity are associated with heart rate increases and relative increases in parasympathetic activity are associated with heart rate decreases. In addition, human lesion and electrical stimulation studies have revealed that the right insula controls cardiac sympathetic activity whereas the left insula is predominantly associated to parasympathetic activity [17]–[19].

Heart rate variability (HRV) is a physiological phenomenon where the time interval between heart beats varies. It is measured by the variation in the beat-to-beat interval. HRV is a simple and non-invasive quantitative marker of autonomic function. As a result of continuous variations of the balance between OS and PS neural activity influencing heart rate, intervals between consecutive heartbeats (RR intervals) show spontaneously occurring oscillations. For HRV analysis, a Fourier-based spectral analysis is performed of the beat to beat intervals, yielding two main frequencies: a low frequency range (LF: 0.05–0.15 Hz) and a high frequency range (HF 0.15–0.4 Hz) [20]. The high frequency component of HRV is believed to be influenced by vagal activity and is also related to the frequency of respiration [21]. Low-frequency (LF) power is modulated by baroreceptor activities and fluctuations in heart rate in the LF range reflect OS as well as PS influences. Low-frequency power, therefore, cannot be considered to reflect selective OS activity. However if normalized units of LF and HF are considered, the OS and PS influences respectively are emphasized [20]. In HRV frequency domain, normalized units of LF and HF components therefore reflect OS and PS influences respectively.

In two recent PET studies it was demonstrated that inducing a certain amount of stress, HRV correlates positively with activity in the anterior cingulate cortex, caudate nucleus, insula, medial prefrontal cortex extending into the dorsal prefrontal cortex [22], [23]. These areas are also involved in tinnitus related distress [9]. Using similar PET studies, the neural correlates of the HF component (PS) have been delineated as the caudate nucleus, periaqueductal gray and left mid-insula [23], while in fMRI the HF component correlates positively with activity in the hypothalamus, amygdala and anterior hippocampal area, dorsomedial/dorsolateral prefrontal cortex and negatively with the cerebellum, parabrachial nucleus/locus coeruleus, periaqueductal gray, posterior parahippocampal area, thalamus, posterior insular and middle temporal cortices [24]. The left inferior part of the pregenual anterior cingulate cortex also correlates with the HF component of the HRV [25]. The increased LF/HF-ratio (in rectal distension) is correlated with activity in the bilateral insula, putamen, thalamus, midbrain, pons, and cerebellum [26].

The aim of the present study is to investigate the relationship between tinnitus distress and the autonomic nervous system and find out which cortical areas are involved in the autonomic nervous system influences in tinnitus distress by the use of source localized resting state electroencephalogram (EEG) recordings and electrocardiogram (ECG).

Quantitative analysis of EEG is a low-cost and useful neurophysiological approach to study the brain physiology and pathology [27]. Cortical sources of the EEG rhythms were estimated by standardized low-resolution brain electromagnetic tomography (sLORETA) [28]. sLORETA is a functional imaging technique estimating maximally smoothed linear inverse solutions accounting for distributed EEG sources within Montreal Neurological Institute (MNI) space [28]. This feature is of special importance for the comparison of EEG results with those of most structural and functional neuroimaging studies. sLORETA has been successfully used in recent EEG studies on tinnitus [29]. In this study we investigate which brain areas are involved in tinnitus distress and in the autonomic nervous system control of the distress.

## Materials and Methods

### Participants

Twenty-one patients (N = 21; 15 males and 6 females) with a mean age of 47.44 (*Sd* = 12.72 were selected from the multidisciplinary Tinnitus Research Initiative (TRI) Clinic of the University Hospital of Antwerp, Belgium. Tinnitus lateralization and tinnitus type was verified by asking the patient in which ear they perceived the tinnitus and whether they perceived a tone or a noise-like sound. Six patients presented with unilateral tinnitus and 15 patients with bilateral tinnitus. Nine patients perceived a pure tone phantom sound and 16 patients a narrow band noise (hearing a noise-like tone within a certain frequency range). No patients included in the study perceived their tinnitus centrally in the head. Individuals with pulsatile tinnitus, Ménière’s disease, otosclerosis, chronic headache, neurological disorders such as brain tumors, and individuals being treated for mental disorders were not included in the study.

Participants were requested to refrain from alcohol consumption 24 hours prior to recording, and from caffeinated beverages consumption on the day of recording. Patients were also given the validated Dutch version of the Tinnitus Questionnaire [30] originally published by Goebel and Hiller [31]. Goebel and Hiller described this TQ as a global index of distress and the Dutch version was further confirmed as a reliable measure for tinnitus-related distress [30], [32]. At the moment of the study patients did not take any pharmacological agent.

This study was approved by the local ethical committee (Antwerp University Hospital) and was in accordance with the declaration of Helsinki. Written informed consent was obtained from all patients.

### EEG/ECG Data Collection

Recordings (Mitsar-201, NovaTech http://www.novatecheeg.com/) were obtained in a fully lighted room with each participant sitting upright on a small but comfortable chair. The actual recording lasted approximately 5 min. The EEG was sampled with 19 electrodes (Fp1, Fp2, F7, F3, Fz, F4, F8, T7, C3, Cz, C4, T8, P7, P3, Pz, P4, P8, O1 O2) in the standard 10–20 International placement referenced to linked ears and impedances were checked to remain below 5 kΩ. Two ECG electrodes were place on the heart axis. EEG and ECG were measured for 5 minutes. Data were collected eyes-closed (sampling rate = 1024 Hz, band passed 0.15–200 Hz). To minimize respiratory influences on HRV, respiration is controlled at 12 beats per minute using auditory cues (i.e. tone 1000 Hz). We selected auditory cues as this is the standard method when collecting ECG data during eyes closed EEG [33]. No patients indicate that this auditory cue interfered with the tinnitus perception or auditory attention to the tinnitus.

### EEG Analysis

Data were resampled to 128 Hz, band-pass filtered (fast Fourier transform filter) to 2–44 Hz and subsequently transposed into Eureka! Software [34], plotted and carefully inspected for manual artifact-rejection. All episodic artifacts including eye blinks, eye movements, teeth clenching, or body movement were removed from the stream of the EEG. Average Fourier cross-spectral matrices were computed for bands delta (2–3.5 Hz), theta (4–7.5 Hz), alpha (8–12 Hz), low beta (13–21 Hz), high beta (21.5–30 Hz) and gamma (30.5–44 Hz) [28], [35].

### ECG Analysis

ECG signals are processed by frequency domain methods as recommended by the Task force [20]: QRS complexes are recognized from the short-term artifact-free ECG recordings from which peaks (R-waves) are detected and from which intervals between two consecutive peaks (RR intervals) are calculated. Once HRV time series are extracted they are analyzed in frequency domain using HRV Analysis Software 1.1 for windows developed by The Biomedical Signal Analysis Group, Department of Applied Physics, University of Kuopio, Finland (see http://kubios.uku.fi/) and generating low frequency (LF: 05–.15 Hz ) and high frequency (HF:.15–.40 Hz). Also the LF/HF-ratio was calculated.

### Source Localization

Standardized low-resolution brain electromagnetic tomography (sLORETA) was used to estimate the intracerebral electrical sources that generated the scalp-recorded activity in each of the eight frequency bands [28]. sLORETA computes electric neuronal activity as current density (A/m^2^) without assuming a predefined number of active sources. The sLORETA solution space consists of 6239 voxels (voxel size: 5×5×5 mm) and is restricted to cortical gray matter and hippocampi, as defined by digitized MNI152 template [36].

The tomography sLORETA has received considerable validation from studies combining LORETA with other more established localization methods, such as functional Magnetic Resonance Imaging (fMRI) [37], [38], structural MRI [39], Positron Emission Tomography (PET) [40]–[42]. Further sLORETA validation has been based on accepting as ground truth the localization findings obtained from invasive, implanted depth electrodes, in which case there are several studies in epilepsy [43], [44] and cognitive ERPs [45]. It is worth emphasizing those deep structures such as the anterior cingulate cortex [46], and mesial temporal lobes [47] can be correctly localized with these methods.

### Region of Interest Analysis

The log-transformed electric current density was averaged across all voxels belonging to the region of interest. Regions of interest were defined based on previous brain research on HRV as well as tinnitus related distress. Regions of interest were respectively the left insula (LI) and right insula (RI)(BA13) [9], dorsal anterior cingulate cortex (BA24 left and right) [9] and subgenual anterior cingulate cortex (BA25 left and right) [9], primary (BA41) and secondary (BA21) auditory cortex [48] and the orbitofrontal cortex (BA10) [12]. Region of interest analyses were computed for the different frequency bands separately.

A lateralization index for the insula was calculated for each frequency band,
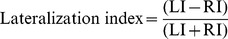
where LI and RI are the log-transformed electrical current density in the left and right insula, respectively. This method is similar to Weisz et al. [49]. Pearson Correlations were calculated and corrections for multiple comparisons for the frequency bands (i.e. Bonferroni) were applied.

### Statistical Analyses

We conducted a whole brain analysis and region of interest (ROI) analyses. Whole-brain analysis is automated and un-biased, making no assumptions about any regions of particular interest. However, this technique requires a great number of subjects to achieve statistical significance and it is possible that smaller changes may not be easily identified. This is one of the reasons why it is common practice to conduct a secondary ROI analysis, testing for statistically significant differences only in the voxels that are deemed of interest by an *a priori* hypothesis. An ROI analysis can be used to corroborate the findings of previous studies, or those obtained during the whole-brain analysis. This is of special importance in studies with a small sample size.

First, correlations are calculated between respectively LF, HF, LF/HF and distress with brain activity (whole brain analysis). The methodology used for the sLORETA correlations is non-parametric. It is based on estimating, via randomization, the empirical probability distribution for the max-statistic, under the null hypothesis comparisons [50]. This methodology corrects for multiple testing (i.e., for the collection of tests performed for all voxels, and for all frequency bands). Due to the non-parametric nature of the method, its validity does not rely on any assumption of Gaussianity [50]. sLORETA statistical contrast maps were calculated through multiple voxel-by-voxel comparisons in a logarithm of F-ratio. The significance threshold was based on a permutation test with 5000 permutations.

Secondly, Pearson correlations are calculated between the lateralization index of the insula and LF/HF-ratio. Based on these findings we conducted a third step, a multivariate ANOVA with LF/HF-ratio and TQ as dependent variables and the lateralization index of the insula in both the alpha and gamma band as independent variables. The reason to include these frequency bands was that both frequency bands correlated with the LF/HF-ratio.

In addition a Pearson correlation analysis was conducted between the dorsal, subgenual and pregenual anterior cingulate cortex, primary and secondary auditory cortex and the orbitofrontal cortex with the respectively the LF, HF, LF/HF-ratio and the TQ, to validate previously obtained results [9], [10].

Lastly, we applied a median-split on both the TQ and the LF/HF-ratio. A median split [51] is a data driven post-hoc stratification that allows us to test the group difference between a low versus high TQ (i.e. distress) and low and high LF/HF-ratio. We applied an ANOVA with TQ (low vs. high) and LF/HF-ration (low vs. high) as independent variables and log-transformed current density at the pregenual anterior cingulate cortex for respectively the high beta and gamma band as dependent variable. We opt to do this analysis for the pregenual anterior cingulate cortex as region of interest for these specific frequency bands, as both frequencies correlated with the TQ and LF/HF ratio.

## Results

### Tinnitus Questionnaire

The mean TQ was 39.22 (Sd = 15.13). No correlation could be found between the TQ and respectively LF, HF and LF/HF-ratio HRV.

### Whole Brain and HRV

#### 1. LF-HRV

**Figure 1:**
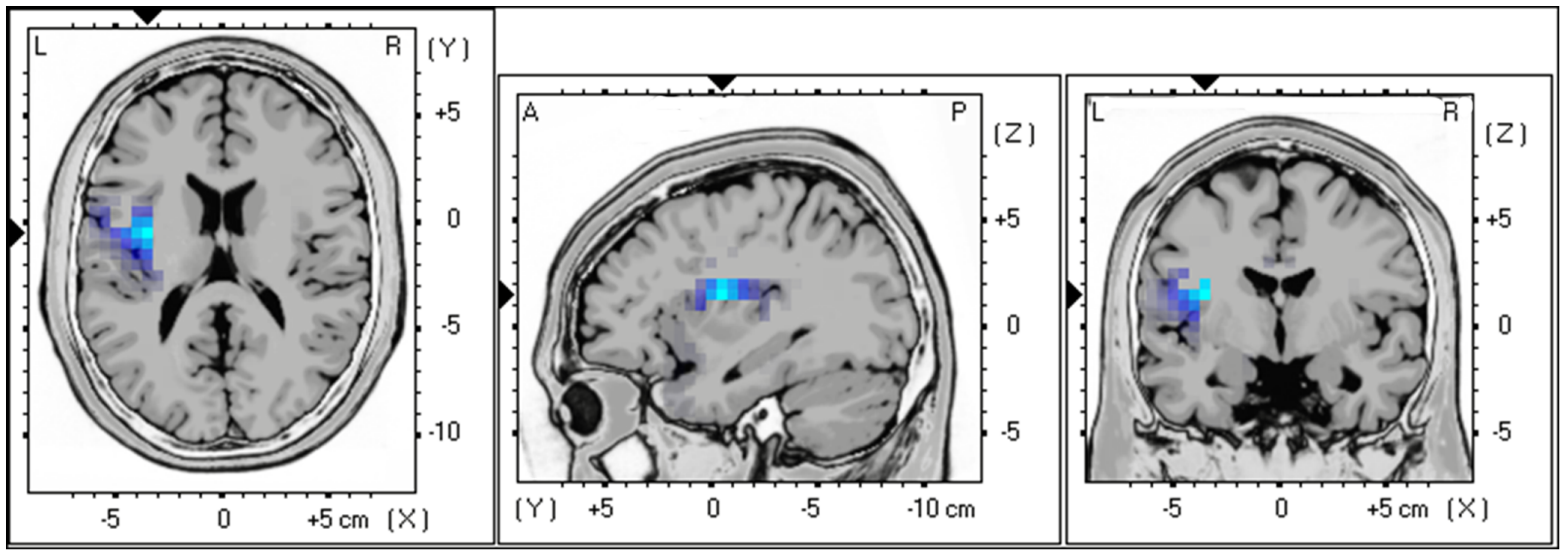
Negative correlation between LF (sympathetic+parasympathetic) HRV and Alpha activity in the left insula (BA13), indicating that decreased alpha activity in the left insula goes together with increased LF-HRV.

Analysis of LF-HRV (a combination of sympathetic and parasympathetic activity) and the left insula (BA13) for alpha activity revealed a significant negative correlation (r = −.42, *p*<.05) indicating that decreased alpha activity in the left insula goes together with increased LF-HRV (see Figure 1).

No significant correlation could be retrieved in delta, theta, low beta, high beta and gamma frequency bands.

#### 2. HF-HRV

**Figure 2:**
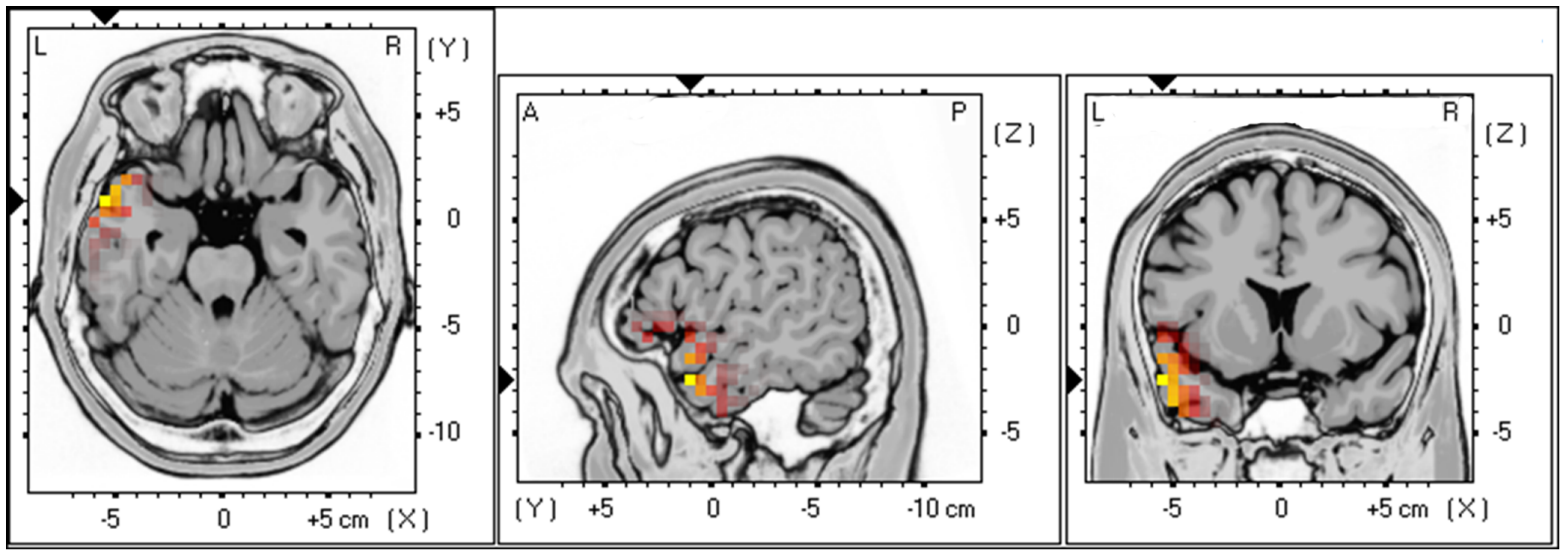
Positive correlation between HF (parasympathetic) HRV and Alpha activity in the rostral portions of the superior temporal gyrus and the middle temporal gyrus (BA21/38). That is increased activity in the rostral portions of the superior temporal gyrus and the middle temporal gyrus goes together with increased HF-HRV.

Results yielded as a significant positive correlation (r = .68, *p*<.05) between HF-HRV (i.e. sympathetic activity) and rostral portions of the superior temporal gyrus and the middle temporal gyrus (BA21/38) for alpha activity (see Figure 2). That is increased activity in the rostral portions of the superior temporal gyrus and the middle temporal gyrus goes together with increased HF-HRV.

No significant correlation could be retrieved in delta, theta, low beta, high beta and gamma frequency bands.

#### 3. LF/HF-ratio

**Figure 3:**
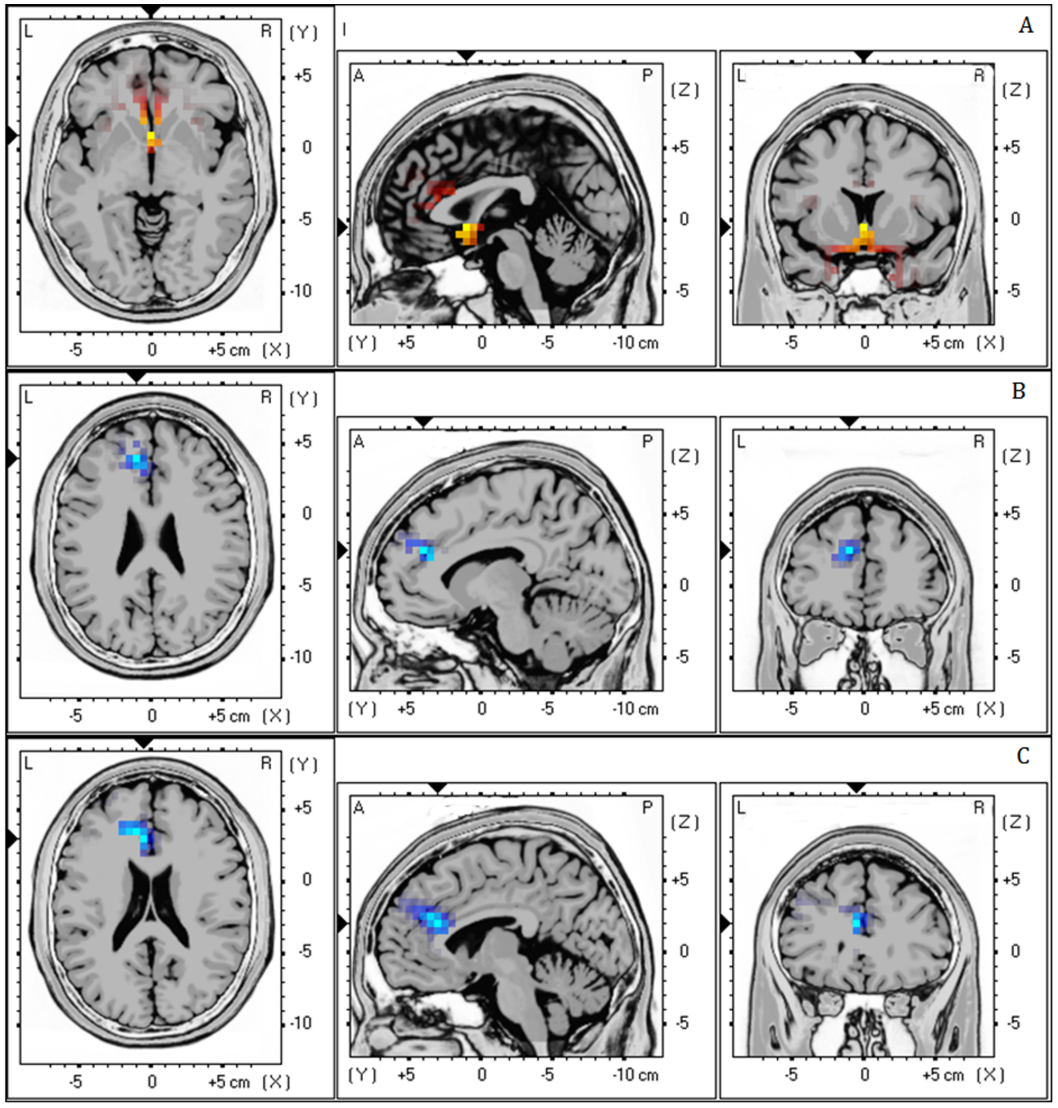
(a) Positive correlation between LF/HF-ratio (sympathetic/parasympathetic ratio) HRV and theta activity in the subgenual anterior cingulate cortex (BA25) (r = .43, *p*<.05); (b) & (c) Negative correlation between LF/HF-ratio HRV and High Beta (r = −.45, *p*<.05) and Gamma activity (r = −.46, *p*<.05) in the left pregenual anterior cingulate cortex (BA24) extending into dorsal lateral prefrontal cortex (BA9).

Analysis demonstrated a positive correlation between LF/HF-ratio (i.e. high numbers mean dominance of sympathetic activity while low numbers mean dominance of the para-sympathetic activity) and Theta activity (r = .43, *p*<.05) in the subgenual anterior cingulate cortex (BA25) (see Figure 3a). This correlation indicates that increased activity in the subgenual anterior cingulate cortex goes together with increased LF/HF-ratio. Also a negative correlation was revealed between LF/HF-ratio HRV and high Beta (r = −.45, *p*<.05) and Gamma (r = −.46, *p*<.05) activity in the pregenual anterior cingulate cortex (BA24) extending into dorsal lateral prefrontal cortex (BA9) (see Figure 3b&c). This latter correlation shows that decreased activity in the pregenual anterior cingulate cortex goes together with increased LF/HF-ratio. No significant correlation could be retrieved in delta, theta and low beta frequency bands.

### Whole Brain and Distress

**Figure 4:**
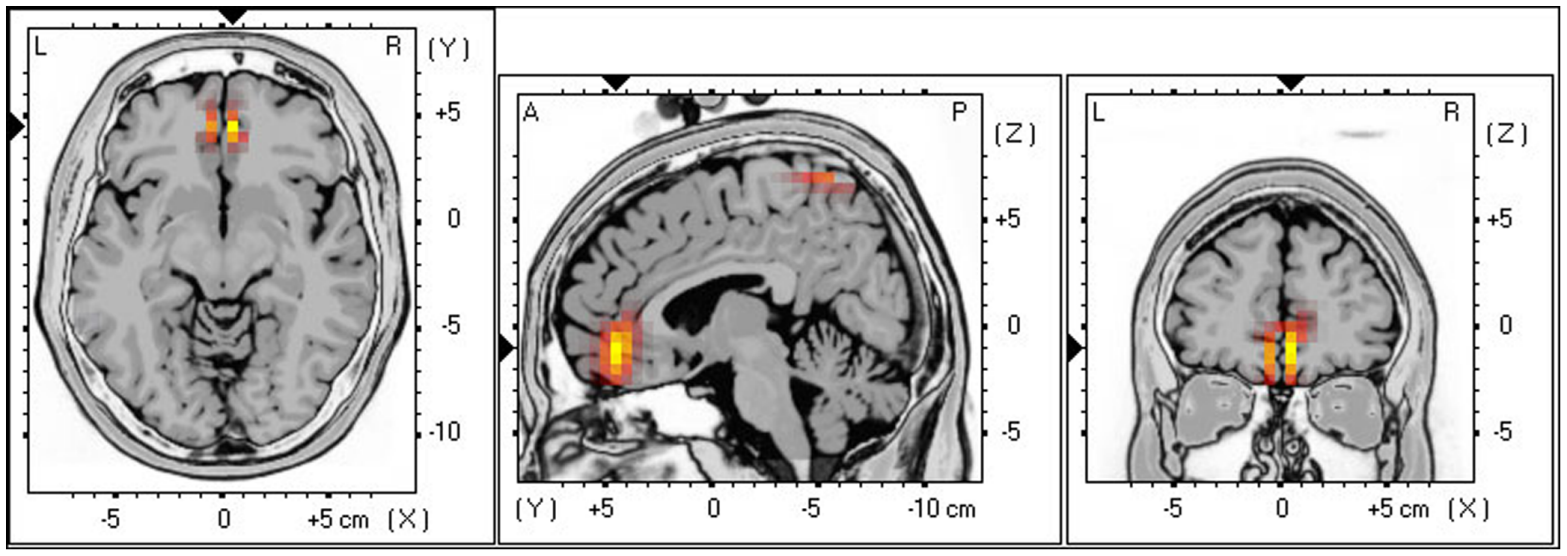
A positive correlation between the distress as measured with the TQ and the whole brain demonstrated a significant effect for the pregenual/subgenual anterior cingulate cortex (BA24/25) (r = .42, *p*<.05) for the alpha frequency band.

A correlation analysis between the distress as measured with the TQ and the whole brain demonstrated a significant effect for the pregenual/subgenual anterior cingulate cortex (r = .42, *p*<.05 (see Figure 4) for the alpha frequency band. This correlation indicates that increased activity in the anterior cingulate cortex goes together with increased distress. No significant correlation could be retrieved in delta, theta, low and high beta and gamma frequency bands.

### Region of Interest Analysis

#### 1. Lateralisation index

**Figure 5:**
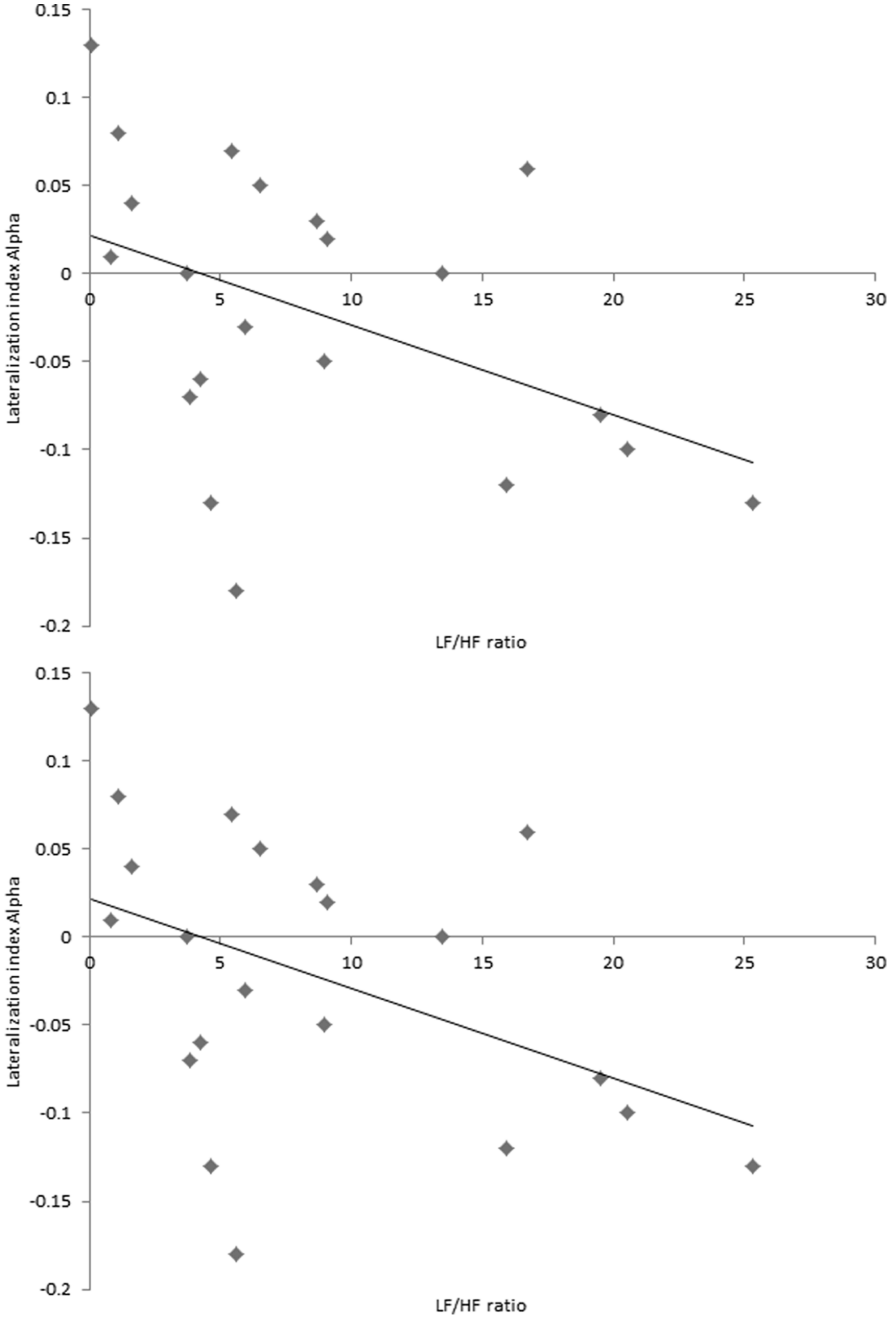
Scatterplots and regression lines for respectively the lateralization index of the insula for Alpha and LF/HF-ratio. Significant negative correlation were found between lateralization index of the insula for Alpha activity and LF/HF-ratio (r = −.45, *p*<.05) and Gamma activity and LF/HF-ratio (r = −.43, *p*<.05).

Significant negative correlations were found between lateralization index of the insula for Alpha activity and LF/HF-ratio (r = −.45, *p*<.05) and Gamma activity and LF/HF-ratio (r = −.43, *p*<.05) (see figure 5). In addition, a significant negative correlation was found between lateralization index of the insula for Alpha activity with TQ (r = −.48, *p*<.05). No significant effects were obtained for the delta, theta, low and high beta frequency bands.

A multivariate ANOVA revealed that both for the lateralization index of the insula for alpha activity (*F*(2,17) = 11.48, *p*<.001) and gamma activity (*F*(2,17) = 4.11, *p*<.05) could be associated with the TQ and LF/HF–ratio (see figure 5). A test of between-subjects effects further revealed that the alpha lateralisation index could be associated with both TQ (*F*(1,20) = 5.69, *p*<.05) and LF/HF-ratio (*F*(1,20) = 6.01, *p*<.05), while the gamma lateralisation index could be associated only with LF/HF-ratio (*F*(1,20) = 3.82, *p*<.05) and no TQ (*F*(1,20) = .52, *p* = .45).

#### 2. LF-HRV, HF-HRV and LF/HR-ratio

A correlation between the LF-HRV, HF-HRV and LF/HR-ratio and respectively the dorsal, pregunal and subgenual anterior cingulate cortex, primary and secondary auditory cortex and the orbitofrontal cortex was computed. This analysis revealed as significant effect for LF/HF with respectively the subgenual anterior cingulate (r = .43, *p*<.05) for the theta frequency band, the pregenual anterior cingulate cortex for the high beta (r = −.45, *p*<.05) and gamma (r = −.46, *p*<.05) frequency band. No significant effects were obtained for primary and secondary auditory cortex and the orbitofrontal cortex.

#### 3. TQ

A significant correlation was obtained for the dorsal anterior cingulate cortex and the TQ for High Beta activity (r = .34, *p*<.05), while the subgenual anterior cingulate cortex was correlated with Alpha activity (r = .34, *p*<.01). No significant effects where obtained for the other frequency bands.

No significant effects could be obtained for the orbitofrontal cortex and the primary and secondary auditory cortex.

#### 4. Interaction between HRV and TQ

**Figure 6:**
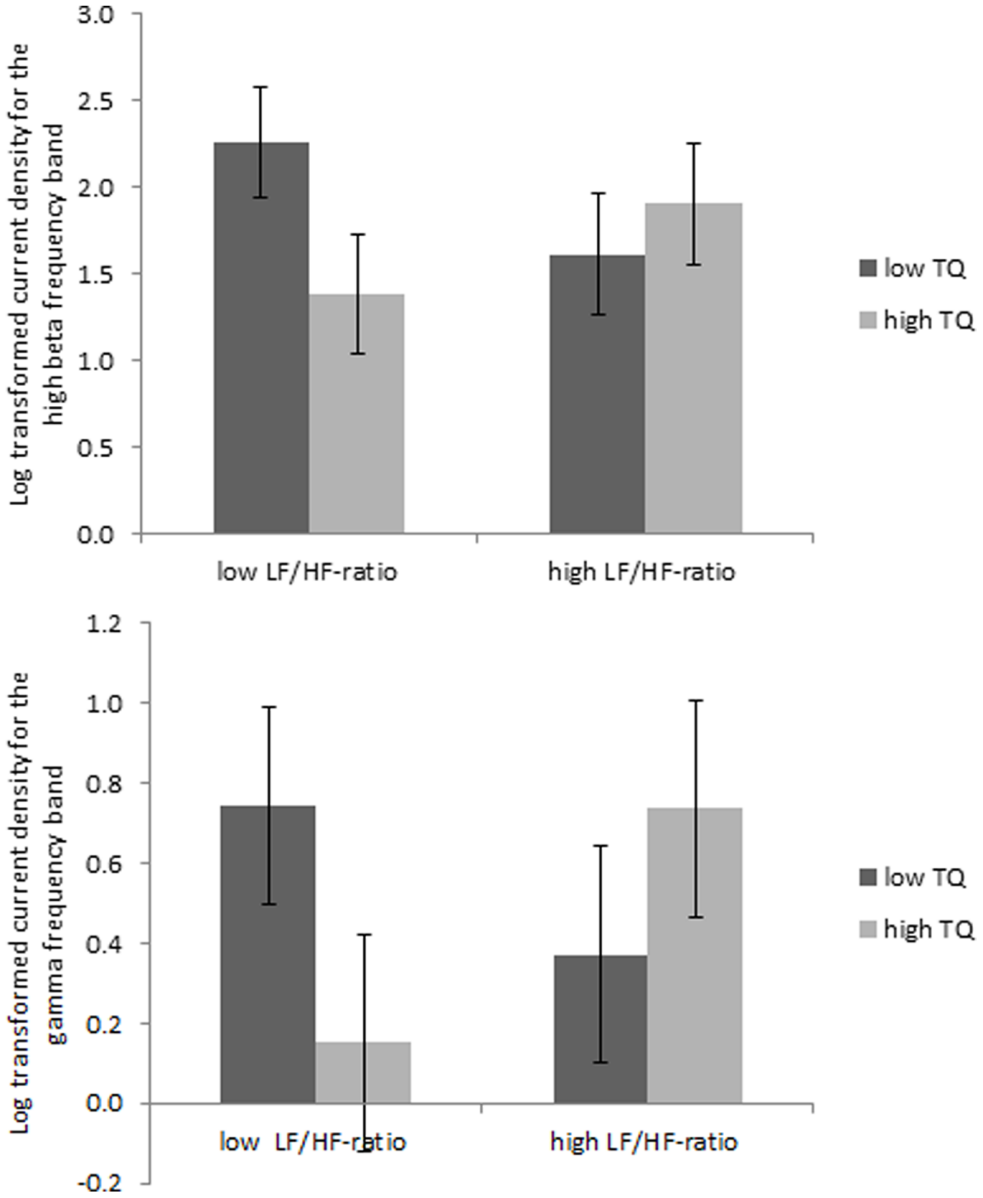
A marginal significant interaction effect between distress (low vs. high) and LF/HF-ratio (low vs. high) for the pregenual anterior cingulate cortex in the high beta (*F* = 2.93, *p = *.10) and gamma (*F* = 3.27, *p = *.09) frequency band.

A marginal significant interaction effect (see figure 6), was obtained between distress (low vs. high) and LF/HF-ratio (low vs. high) for the pregenual anterior cingulate cortex in the high beta (*F* = 2.93, *p = *.10) and gamma (*F* = 3.27, *p = *.09) frequency band. A detailed analysis demonstrates that patients with a low LF/HF ratio and high distress have a lower current density with these specific frequency bands in the pregenual anterior cingulate cortex in comparison to patients with a low LF/HF ratio and high distress. No difference was found in comparison to patients with high LF/HF ratio irrespective of low or high distress.

No significant effects were obtained for the delta, theta, alpha and low beta frequency bands.

## Discussion

The autonomic nervous system is controlled by the sympathetic and parasympathetic system. The cardiac sympathetic/parasympathetic or sympathovagal balance is reflected by the LF (OS+PS)/HF (PS) ratio [52]. Previous research already revealed the relationship between the autonomic nervous system and specific brain regions such as the subgenual and dorsal anterior cingulate cortex and insula. Interestingly, these brain areas are also involved in tinnitus related distress [9]. The aim of the present study is to investigate the relationship between tinnitus distress and the autonomic nervous system and find out which cortical areas are involved in the autonomic nervous system influences in tinnitus distress.

### Heart Rate Variability and the Brain

A negative correlation was demonstrated between LF/HF-ratio and the pregenual anterior cingulate cortex extending into the dorsal lateral prefrontal cortex for respectively high beta and gamma activity. That is, decreased LF and/or increased HF goes together with an intensification of high beta and gamma activity in the pregenual anterior cingulate cortex. A positive correlation has previously been found between neural activity in the pregenual anterior cingulate cortex and the parasympathetically linked HF component of heart rate variability in an anxious population [22] and when performing a Stroop task [25]. Together with the pregenual anterior cingulate also the dorsal lateral prefrontal cortex was involved with regulating LF/HF-ratio. This was similarly to previous research indicating that during social threat the dorsal lateral prefrontal cortex activity appears reduced in social phobia compared to controls [53]. The pregenual anterior cingulate cortex extending into the ventro-medial prefrontal cortex predominantly mediates parasympathetic control [54]–[56], and the ventromedial prefrontal cortex inactivates sympathetic activity [57], suggesting that the ventromedial prefrontal cortex exerts an predominantly parasympathetic modulation of the sympathovagal balance. This is in accordance with the positive correlation found between LF/HF-ratio and the subgenual anterior cingulate cortex for theta activity, revealing that increased LF and decrease HF goes together with an increase of theta activity in the subgenual anterior cingulate cortex. A fMRI study also related HRV and sympathetic cardiac influence with the subgenual anterior cingulate cortex [58]. It has also been shown that increased activity in posterior subgenual anterior cingulate cortex extending into nucleus accumbens-ventral tegmental area is involved in processing of aversive sounds [59] and unpleasant music [60] and this area has been implicated in mediating limbic-autonomic interactions in tinnitus as well [61], [62]. This area in animals has been considered a visceromotor cortex, due to its connections with the parasympathetic nucleus tractus solitarius [63] and the sympathetic areas in the periaquaductal grey [64]. Furthermore it is functionally connected to the insula and anticorrelated to the dorsal anterior cingulate cortex [65]–[67]. Dorsal anterior cingulate activity covaries with blood pressure, emotional heart rate changes, cardiac sympathetic tone and pupillary changes [68].

Our results further revealed that increased LF goes together with a decrease in activity in the left insula. Furthermore tinnitus distress correlates negatively with the lateralization index of the insula for alpha, revealing that increased tinnitus distress is associated with a decrease in the lateralization index in the insula. These findings are in accordance with previous research revealing that increased insular activity is associated with subjective emotional and bodily awareness, as well as interoception [69]. The insula has been implicated in autonomic nervous system control [20], [68], [70], [71] and might therefore be related to the autonomic components involved in distress [72], [73], induced by the phantom sound. In a recent study it was further revealed that the insula is involved in pain sensitivity [74]. In addition, a region of interest analysis revealed that LF/HF-ratio correlates negatively with the lateralization index for alpha and gamma activity in the insula. This latter result demonstrates that increased LF and(or) decreased HF goes together with a decrease of activity in the left insula and an increase of activity in the right insula.

### Distress and the Brain

Tinnitus distress, as reflected by the TQ, correlates positively with the lateralization index of the insula in alpha, indicating that an increase in right insula and/or a decrease in left insula go together with an increase in tinnitus related distress. It has already been shown that alpha activity in both the left and right insula correlates with the severity of tinnitus-related distress [9]. In addition also a correlation was found between TQ and the dorsal anterior cingulate cortex for high beta frequency band and the TQ and subgenual anterior cingulate cortex for alpha frequency band [9].

### Heart Rate Variability and Distress

No correlation could be found between the TQ and respectively LF, HF and LF/HF-ratio HRV. This is similar to previous research demonstrating no correlation between trait anxiety and LF, HF and LF/HF-ratio HRV [75]. However research further revealed that the lateralisation index for alpha activity can be associated with both TQ and LF/HF-ratio, while the lateralisation index for gamma could only be associated to the LF/HF-ratio. Taking these findings together this would suggest that the left and right insula in alpha activity influence the TQ and LF/HF-ratio.

### Heart Rate Variability, Distress and the Brain

Our results seem to indicate that the sympathovagal balance is controlled by subgenual and pregenual anterior cingulate cortex, whereas the left and right insula control parasympathetic and sympathetic activity respectively. Interestingly, the tinnitus distress correlates positively with the lateralization index of the insula, indicating that distress seems to be sympathetically mediated, as has been demonstrated previously [76]. In addition we found an interaction effect between the LF/HF ratio and distress for the pregenual anterior cingulate cortex within high beta and gamma frequency band. It has been proposed [77] and shown [81] that the pregenual anterior cingulate cortex mediates a the top-down inhibitory effect on tinnitus [78], analogous to what has been shown for pain [79]–[81]. This top-down, descending pain inhibitory system involves the anterior insula, pregenual anterior cingulate cortex, and periaqueductal gray [81]. In the auditory system it involves the subgenual and pregenual anterior cingulate cortex [62], [78], [82], and could also involve the anterior insula and the longitudinal tectal column, a recently discovered structure, adjacent to the periaqueductal gray, the auditory analogue of the somatosensory periaqueductal gray [77]. In pain, fluctuations of activity in the dorsal anterior cingulate cortex and anterior insula determine whether a near threshold pain stimulus is consciously perceived or not [83], and the same holds for an auditory stimulus [84]. Thus by decreasing the anterior cingulate and anterior insular activity painful and auditory stimuli can be processed without the stimuli reaching awareness, linked to the anterior insula [85]–[87].

### Limitations of the Study

One major limitation of this and any EEG based approach is that no subcortical activity can be analyzed, limiting network description to cortical sources. The data presented should therefore be viewed acknowledging this limitation. Another limitation of the present study is that no control group was included in the study. However, our analysis shows that within tinnitus patients certain brain areas play an important role in the central control of HRV and that these brain areas are also correlated to tinnitus related distress. This does not exclude that for control subjects this could also be the matter. Yet previous research has shown that dorsal anterior cingulate cortex and the subgenual anterior cingulate cortex show increased activity in respectively the Beta and Alpha activity in comparison to a control group [9], [10]. Hence, we think that the results obtained in our study might be reliable and valid, but it should be stressed that further research confirming and extending these results is needed, e.g. by using a control group.

### Conclusion

In conclusion our data suggest that the dorsal and subgenual anterior cingulate cortex, as well as the left and right insula are important in the central control of heart rate variability in tinnitus patients. Whereas the sympathovagal balance is controlled by the subgenual and pregenual anterior cingulate cortex, the right insula controls sympathetic activity and the left insula the parasympathetic activity. The perceived distress in tinnitus patients seems to be sympathetically mediated.
